# 

**Published:** 1886-02

**Authors:** 


### EDITORIAL.

### Mississippi Valley Association Dental Surgeons.

The forty-second annual meeting of this time-honored Society will take place in the lecture room of the Ohio Dental College, Wednesday morning at 10 o'clock, March 3d, and continue in session for three days. It goes without saying that this is one of the best Societies ever organized by the dental profession, and it behooves us to sustain it with all our might. Many of the great ones in the country graduated from its councils, and have gone forth to spread their knowledge abroad for the benefit of the younger ones growing up in the ranks, as well as for the good of the public at large.

One of the characteristics of the American people, is their ability to "take hold" of a thing and make a success of it, even in the face of apparently insurmountable obstacles. Now, we want every body who reads these lines, to take hold with a vengeance, prepare a short paper upon any subject pertaining to the theory or practice of dentistry, bring with him any new instrument or appliance he may have invented or improved upon, collar one or more of his professional neighbors, and arm in arm walk into the assembly room promptly on time, cocked and primed, and ready to begin business at the first tap of the gavel.

There is a large number of practitioners in Southern Ohio, Northern Kentucky, Western Indiana, and, in fact, all through these States, not to mention those adjoining, who do not attend their State Societies, and who have no other good Society so near at hand, and of such easy access as the M. V. D. A. For these reasons they should attend its meetings in large numbers, and come prepared to take an active part in the proceedings. We are often told that it is not necessary to attend, as they can get reports of the meetings in the journals; very true, but the reports so made are not for that purpose at all, they merely put on record what has been said and done, but cannot convey the full thought of a speaker, for they lack the ring of the voice, and the flash of the eye that makes one always remember a speech of Atkinson, Rawls or McKellops.

It is not necessary to enlarge upon this theme, though it will stand it, for the good sense of all will prompt them without further urging to come to the meeting in large numbers.

CHICAGO, Jan. 7th, 1886.

The Executive Committee of the Illinois State Dental Society announces the following list of Reports and Essays for the next Annual Meeting, which will be held at Rock Island, the second Tuesday in May.

1. Report of the Committee on Dental Science and Literature.
   Dr. Homer Judd, of Alton, (Chairman),
   Dr. C. R. E. Koch, of Chicago,
   Dr. M. L. Hanaford, of Rockford.
2. Report of the Committee on Dental Art and Mechanism.
   Dr. J. Frank Marriner, of Ottowa, Chairman.
   Dr. J. A. Swasey, of Chicago,
   Dr. W. T. Magill, of Rock Island.
3. Essay, Dr. John S. Marshall, of Chicago, "Oral Surgery."
4. Essay, Dr. C. R. Taylor, of Streator,
   "Preparation of Pulp Canals and Cavities for Filling."

5. Essay, Dr. Homer Judd, of Alton.
"The Retention of Pulpless Teeth in the Jaw."
6. Essay, Dr. J. D. Moody, of Mendota, "Post Graduate Study."
7. Essay. Dr. J. G. Reid, of Chicago, "Oral Chemistry."
8. Essay, Dr. E. S. Talbot, of Chicago.
"Separating Teeth preparatory to Filling them."

It is probable that Dr. G. V. Black, of Jacksonville, may have an Essay, the subject of which he is not yet ready to announce.

The Executive Committee have decided to issue this preliminary announcement (which is subject to such changes or additions as may be found desirable), in order to give members of the Society an opportunity to prepare themselves for a profitable discussion of the subjects proposed, and to avoid the necessity for issuing the final programme till very near the time for the meeting.

EDMUND NOYES,
E. J. GREEN,
W. H. TAGGART,
*Executive Committee.*

### Mississippi Valley Association of Dental Surgeons.

The forty-second annual meeting of the Mississippi Valley Association of Dental Surgeons, will take place in the lecture room of the Ohio Dental College, at 10 a. m., Tuesday, March 2d, 1886.

For this meeting of the Association there will be papers on the several subjects on the programme by the gentlemen there named, thus enabling us to have the subject for discussion promptly brought before each session. We feel satisfied that papers presented by these gentlemen will insure a large and interesting meeting. A most cordial invitation is extended to all members of the profession to come and participate, feeling assured that they will be interested and profited by the papers and the discussion thereof. Those having new methods and appliances, please bring them for the Clinic.

PROGRAMME.

I. Treatment of Pulpless Teeth, J. W. Jay, Richmond, Ind.

II. Some Phenomena observed in the use of Antiseptics in Oral Surgery, H. A. Smith, Cincinnati, O.

III. Bridge Work, J. E. Low, Chicago, Ill.

IV. Clinic; Bridge Work; Artificial Crowns; Herbst's Method, Dental Appliances, etc.

V. Report of a case of death by the lodgement of a partial Denture (gold) in the Bronchial Tube, J. W. Joy, Richmond, Ind.

VI. Amalgam Fillings from the stand-point of to-day, C. M. Wright; F. A. Hunter, Cincinnati, O.

"OFFICERS."

President, A. F. Emminger.
First Vice-President, E. G. Betty.
Second Vice-President, Otto Arnold.
Recording Secretary, A. G. Rose.
Treasurer, F. A. Hunter.
Corresponding Secretary, W. D. Kempton.

EXECUTIVE STANDING COMMITTEES.

C. H. James, N. L. Moore, M. N. Fletcher.

MEMBERSHIP.

F. W. Sage, J. S. McCampbell, J. R. Callahan.

PUBLICATION.

J. Taft, O. N. Heise, J. S. Cassidy.

ETHICS.

C. M. Wright, J. M. Clyde and G. W. Keely.

We regret to learn that Mr. Oakley Coles has taken leave of the profession, of which he has so long been one of the most useful members, to embrace that of the church.

In English dentistry he will ever be remembered as one of that noble band, who, by their persistent and well directed efforts, secured for their profession recognition from the College of Surgeons, and in consequence an enviable position in the ranks of scientific society.

His contributions to the literature of dentistry have been very numerous, both in book form and in communications to the British Odontological Society and the British Dental Association.

His addresses on special occasions have been far above the usual standard of such utterances, to wit the address on dental literature delivered to the Liverpool meeting of the Dental Association in August, 1882. It is without exception the best summary of dental literary history ever written, and the appendix, though incomplete and not devoid of error, omits no work of value.

The following papers were communicated by Mr. Coles, to the Odontological Society of Great Britain :

1869. On the treatment of defects of the palate caused by syphilis.

1871. On the celluloid base.

1872. On the production of articulate sound (speech).

1874. On the condition of the mouth and teeth during pregnancy.

1878. On the so-called Riggs disease.

1880. An attempted classification of Deformities of the upper jaw.

In addition to the above, his untiring industry has been manifested in numerous papers which have appeared in the *British Journal of Dental Science* and in the *Monthly Review of Dental Surgery* of which latter journal Mr. Coles was the founder and some time editor.

Deformities of the mouth, 8vo., London, 1870; 2nd edition 1870; 3rd edition 1881. First American edition, 8vo., Philadelphia, 1868; 2nd American edition 1870; 3rd American edition 1881.

The teeth notes, on their pathology, 8vo., London, 1872.

A manual of dental mechanics, 8vo., London, 1873; 2nd. edition 1876. First American edition, 8vo., Philadelphia, 1873.

The same in French, 8vo., Paris, 1874.

On the condition of the mouth and teeth during pregnancy, 8vo., London, 1874.

The dental student's note book, 8vo., London, 1876. First American edition, 8vo., Philadelphia; 2nd edition 1880.

### Ninth International Medical Congress.

### SECTION ON DENTAL AND ORAL SURGERY.

As there seems to be some misapprehension in the minds of some, and earnest enquiry by others as to the status of the Section on Dental and Oral Surgery in the International Medical Congress to be held in Washington, D. C., in 1887; it seems right and proper that some statement be now made in regard to the organization and progress of the work.

It is very generally known that the section has been established and organized; the following officers have been appointed; viz.: a President, one Vice-President and two Secretaries.

Fourteen gentlemen of recognized ability and high professional standing, from various parts of the country have accepted position upon the Council, and have pledged themselves to do all they can to make this Section a success. At the next meeting of the Executive Committee, ten or twelve more names will be added to the Council, as may seem best. Much of the preliminary work in arranging the matters of the Section has been in the main accomplished.

A program embracing subjects of great interest to the profession of the world, has been outlined and is now under consideration; and as soon as completed, the secretaries will open correspondence with the eminent men of the profession in Europe and America, relative to the work to be done. Quite a number have already indicated a desire to prepare papers, or at least take some part in the work.

We not only expect but are assured that this Section will receive the hearty support and co-operation of dental specialists, both at home and abroad; and with such manifestations, great hope is entertained that the Section will be eminently successful. We ask for it the co-operation of all who have the best interests of our profession at heart.

A circular will ere long be issued by the Executive Committee giving status of the preparatory work for the Congress.

J. Taft, of Section D. O. S.

### Association.

### Received.

The second edition of *Dental Medicine*, a manual of Dental Materia Medica, has just come to hand, too late for notice in this number of the REGISTER, but will receive attention in the March number.

### The Louisiana State Dental Society.

The annual meeting of this body will be held in "Tulane Hall," at N. O., on the 4th, 5th and 6th of March, 1886.

The profession at large is cordially invited.

The Mardi-Gras festivities, together with the Exposition here and now in full blast, as also cheap R. R. rates and mild weather, ought to be sufficient inducements to our brother dentists to pay us a visit

Any information as regards meeting and accommodations, etc., will be cheerfully and promptly furnished, by addressing,

Yours, respectfully,

R. J. FRIEDRICHS,

*Chairman Executive Committee,*

153 Carondelet Street, New Orleans, La.

### Caulk's Dental Annual No. 4, 1885–6. 8 vo. Camden, Del.

We note a continual growth and improvement in this useful publication. The present number provides the profession with much valuable information not available in any other single book. The more noteworthy items are the Directory of Dental Societies, which we regret to see applies only to the United States. Those which exist in the rest of the world are so few that we hope Mr. Caulk will make the list complete next year. Probably the editors of the dental journals published in foreign countries would give the information necessary. It would be a good thing to add some details with reference to the manner of publication of transactions whether by the society itself or in some recognized journal, and to the number of parts of transactions issued. The list of dental periodicals is not quite complete, the omissions however, are not important. In the necrology will be observed two notable names. I. S. Hyatt, the joint inventor of celluloid and J. M. Riggs, whose name will probably now cease to be applied to the disease which is more appropriately known as Pyorrhœa Alviolaris.

The table of the number of dentists in the various States suggest many reflections. What fertile fields for the profession are to be found in Arizona, New Mexico, and Wyoming, containing from two to four dentists. Our graduates can not or certainly should not complain of over-crowding while these vast territories remain practically empty.

Twenty-one patents covering every mechanical branch of dentistry were taken out in 1885. It would be useful to add the foreign patents next year. Last in order, but certainly first in importance comes the collection of dental laws of the United States which shows that fourteen are still without legislative enactments on the subject. We regret that the editor has not added the form of law suggested by the National Association of State Boards of Dental Examiners which in the interests of uniformity deserves general adoption. The low price at which the work is sold is an illustration of the advantage of combining advertisements with useful text as it must be evident that the greater portion of the profit on the work is drawn from that source.

### CONTENTS.

### COMMUNICATIONS.

### THE DENTAL REGISTER,

A MONTHLY MAGAZINE DEVOTED TO THE INTERESTS OF THE

### DENTAL PROFESSION.

*Terms Reduced to* $2.00 *Per Year. Single Copies,* 25 *Cents.*

### EDITED BY PROF. J. TAFT, D.D.S.

—AND—

### Published by SAM'L A. CROCKER & CO., Ohio Dental and Surgical Depot.

The volume commences in January and closes in December.

The REGISTER being issued monthly is always fresh, therefore Indispensable to the practitioner who desires to keep posted up with the new inventions and improvements which are daily developed. Neither expense nor effort will be spared to give value and variety to its contents. Articles will be illustrated with well executed engravings whenever they will add to the interest or assist to explanation.

Its extensive circulation and frequency of issue make it one of the BEST DENTAL ADVERTISING MEDIUMS in the United States.

All subscriptions must begin with January or July.

Local or special notices, five lines or less, will be inserted for $3 each insertion; over five lines, 50 cts. per line.

All advertising bills payable in advance, or at furthest, after the issue of the first number containing the advertisement. All transient advertisements to be paid for in advance.

☞CONTRIBUTIONS SOLICITED.

Exclusively Editorial Communications should be addressed to the Editor.

The latest number of the REGISTER may be ordered with or without other goods at any time, and all letters should be addressed to

**SAM'L A. CROCKER & CO., Ohio Dental and Surgical Depot, Cincinnati, O.**

